# Profiling Circulating and Urinary Bile Acids in Patients with Biliary Obstruction before and after Biliary Stenting

**DOI:** 10.1371/journal.pone.0022094

**Published:** 2011-07-08

**Authors:** Jocelyn Trottier, Andrzej Białek, Patrick Caron, Robert J. Straka, Piotr Milkiewicz, Olivier Barbier

**Affiliations:** 1 Laboratory of Molecular Pharmacology, Centre Hospitalier Universitaire de Québec (CHUQ) Research Center and the Faculty of Pharmacy, Laval University, Québec, Canada; 2 Department of Gastroenterology, Pomeranian Medical University, Szczecin, Poland; 3 Department of Experimental and Clinical Pharmacology, College of Pharmacy, University of Minnesota, Minneapolis, Minnesota, United States of America; 4 Liver Unit and Liver Research Laboratories, Pomeranian Medical University, Szczecin, Poland; Consejo Superior de Investigaciones Cientificas, Spain

## Abstract

Bile acids are considered as extremely toxic at the high concentrations reached during bile duct obstruction, but each acid displays variable cytotoxic properties. This study investigates how biliary obstruction and restoration of bile flow interferes with urinary and circulating levels of 17 common bile acids. Bile acids (conjugated and unconjugated) were quantified by liquid chromatography coupled with tandem mass spectrometry in serum and urine samples from 17 patients (8 men and 9 women) with biliary obstruction, before and after biliary stenting. Results were compared with serum concentrations measured in 40 age- and sex-paired control donors (20 men and 20 women). The total circulating bile acid concentration increases from 2.7 µM in control donors to 156.9 µM in untreated patients with biliary stenosis. Serum taurocholic and glycocholic acids exhibit 304- and 241-fold accumulations in patients with biliary obstruction compared to controls. The enrichment in chenodeoxycholic acid species reached a maximum of only 39-fold, while all secondary and 6α-hydroxylated species –except taurolithocholic acids – were either unchanged or significantly reduced. Stenting was efficient in restoring an almost normal circulating profile and in reducing urinary bile acids.

**Conclusion:**

These results demonstrate that biliary obstruction affects differentially the circulating and/or urinary levels of the various bile acids. The observation that the most drastically affected acids correspond to the less toxic species supports the activation of self-protecting mechanisms aimed at limiting the inherent toxicity of bile acids in face of biliary obstruction.

## Introduction

Biliary stenosis caused by bile duct stones, chronic pancreatitis or tumors, results in bile retention within the ducts and liver. Such obstruction causes numerous disturbances in liver physiology, particularly for bile acid synthesis, transport and metabolism [1], [2]. Bile acids play essential roles in the control of cholesterol homeostasis and dietary lipid absorption, but are cytotoxic at elevated concentrations. Approximately 200 to 600 mg of bile acids are synthesized daily and compensate for the loss of acids eliminated in the bile and excreted in feces [3]. In humans, the circulating bile acids mainly correspond to the primary cholic (CA) and chenodeoxycholic acids (CDCA) (reviewed in Monte M.J. et al. [4]). These acids are conjugated with taurine and glycine to form amidated detergents [4]. Conjugated and unconjugated bile acids sustain a strong enterohepatic recirculation, through which they can be deconjugated in the intestine and converted into the secondary deoxycholic (DCA) and lithocholic acids (LCA). Bile acids reabsorbed in the intestine can then be further modified back in the liver [4]. These modifications correspond to reconjugation with taurine and glycine, or conversion of CDCA and LCA into the 6αhydroxylated acids, hyocholic (HCA) and hyodeoxycholic acids (HDCA), respectively [5], [6], [7].

It has already been known that biliary obstruction results in a marked accumulation of intrahepatic bile acids, which is reflected by a dramatic increase of the total circulating bile acid concentration [2], [3], [8]. These natural detergents are generally considered as cytotoxic at high concentrations (reviewed in Hofmann A.F. [9]), and their accumulation in the cholestatic liver contributes to the disease, notably by promoting apoptosis and necrosis of hepatocytes [9]. However, bile acid toxicity is generally associated with their amphiphilic properties [9], and the balance between hydrophobic and hydrophilic characters differs among the bile acid species [4]. Accordingly, the hydrophobic DCA has been shown to cause more membrane damage than CA and its conjugates [10], [11], whereas DCA, CDCA and LCA have stronger deleterious effects on cell viability and mitochondrial functions than other bile acids [10], [12], [13].

Our understanding of bile acid toxicity during biliary obstruction is currently limited by the fact that only the total circulating bile acid concentration is generally determined, while the composition of this pool is rarely investigated [2], [3], [8]. Similarly, whether the circulating and urinary levels of all bile acid species are equally corrected when bile flow is restored has only received little attention [2], [14]. A comprehensive analysis of the bile acid profile in serum and urine would therefore be required to fully grasp the toxicological effects associated with biliary obstruction and bile flow restoration. Using liquid chromatography coupled with tandem mass spectrometry (LC-MS/MS), 17 bile acid species were quantified in serum and urine samples from 17 patients with biliary obstruction obtained before and after biliary stenting. Furthermore, the pre- and post-stenting serum concentrations of each acid were compared to data from 40 age- and sex-matched non-cholestatic volunteers.

## Results

### Biliary stenosis causes an accumulation of circulating conjugated bile acids

**Figure 1:**
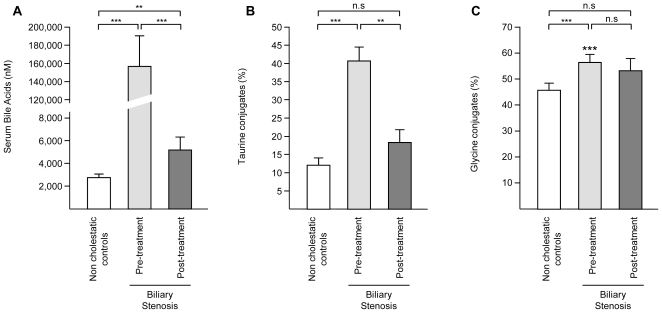
Serum levels of total bile acids (A) and relative abundance of taurine- (B) and glycine- (C) conjugated species in non-cholestatic donors and patients with biliary obstruction before and after biliary stenting. Bile acids were quantified as indicated in the “Experimental Procedures” section. Concentrations (A) and relative abundance of tauro- (B) and glyco-bile acids (C) in biliary stenosis patients (17 donors) before and after stenting were compared to those from a non-cholestatic group (40 volunteers). **A:** Total bile acids: sum of all 17 species. **B:** Percentage of taurine conjugates: sum of taurine-conjugated acids divided by the sum of all bile acids. **C:** Percentage of glycine conjugates: sum of glycine-conjugated acids divided by the sum of all bile acids. Graphics represent the mean ± SEM. *P*-values were determined by the Wilcoxon/Mann-Whitney rank-sum test. **: *p*<0.01, ***:p<0.001. n.s.: not significant.

**Figure 2:**
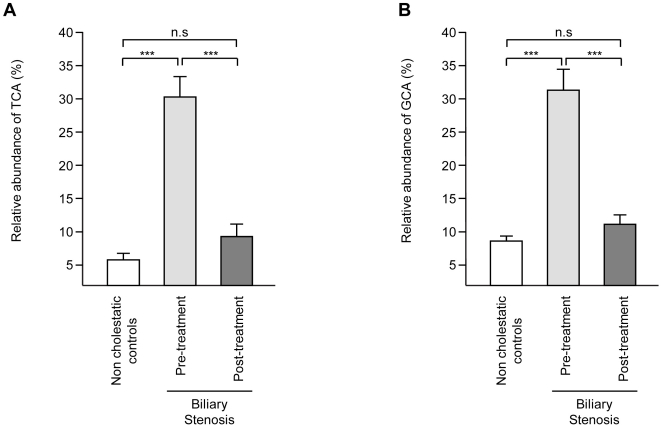
Relative abundance of taurine- (A) and glycine- (B) conjugated cholic acid in serum from non-cholestatic donors and patients before and after biliary stenting. TCA and GCA were quantified as indicated in the “Experimental Procedures” section. Their relative abundance was calculated relatively to the sum of all bile acids. Graphics represent the mean ± SEM (nM). *P*-values were determined by the Wilcoxon/Mann-Whitney rank-sum test. **: *p*<0.01, ***: p<0.001. n.s.: not significant.

The serum profile of bile acids in the non-cholestatic population, used as control in the present study, has been described previously [15]. When compared to this control population, the serum bile acid profile from patients with biliary obstruction exhibited dramatic changes (Table 1). The total of bile acids increased 58-fold (from 2.7 to 156.9 µM (Figure 1A). This accumulation mainly reflected a marked enrichment in total taurine- and glycine-conjugated species (190- and 67-fold, respectively; Table 1). Because tauro-bile acids are lower than glycine conjugates in non-cholestatic samples, their proportion increased from 13 to 42% in non-treated stenosed patients (Figure 1B). In parallel, the relative abundance of glycine conjugates only increased from 46 to 54% (Figure 1C). Actually, the most remarkable changes observed in this population were the 304- and 241-fold accumulation of TCA and GCA, respectively. As a result, these 2 acids accounted for <10% of the total bile acid concentration in non-cholestatic samples, but represented 31 and 32% of bile acid species in patients with obstruction, respectively (Figure 2).

**Table 1:** Serum bile acid composition in non-cholestatic volunteers (A: control) and patients before (B) and after (C) biliary stenting.

				A to B	B to C	A to C
	A: Control	B: Pre-stenting	C: Post-stenting	Change	Change	Change
Bile acids	Mean ± SEM	Mean ± SEM	Mean ± SEM	p Value	p Value	p Value
CDCA	256.8±56.3	84.4±60.2	236.2±62.1	↓:<0.01	↑:<0.05	n.s.
TCDCA	120.2±21.8	16,577.8±4,370.2	286.7±53.3	↑:<0.001	↓:<0.001	↑:<0.01
GCDCA	771.5±111.9	28,534.5±5,754.4	1,986.8±590.4	↑:<0.001	↓:<0.001	↑:<0.01
CA	181.5±83.1	173.3±133.5	86.6±39.4	n.s.	n.s.	n.s.
TCA	179.7±47.0	54,486.9±13,419.0	302.6±82.0	↑:<0.001	↓:<0.001	n.s.
GCA	233.0±56.0	56,222.3±15,714.0	460.4±100.0	↑:<0.001	↓:<0.001	↑:<0.05
UDCA	137.6±25.1	20.3±11.3	1,085.6±446.4	↓:<0.001	↑:<0.001	n.s.
TUDCA	5.0±1.1	74.9±22.4	97.6±30.3	↑:<0.001	n.s.	↑:<0.001
LCA	12.8±1.8	1.5±0.7	26.3±6.2	↓:<0.001	↑:<0.001	n.s.
TLCA	23.4±3.6	11.6±1.8	13.8±2.6	n.s.	n.s.	n.s.
GLCA	16.3±4.1	10.5±2.1	46.7±12.7	n.s.	↑:<0.01	↑:<0.01
LCA-S	7.3±1.1	4.2±1.8	14.3±4.2	↓:<0.01	↑:<0.01	n.s.
DCA	386.7±66.0	20.7±15.7	254.4±99.1	↓:<0.001	↑:<0.01	↓:<0.05
TDCA	44.9±11.8	213.4±70.9	43.2±11.0	↑:<0.001	↓:<0.01	n.s.
GDCA	246.2±42.5	417.6±90.9	225.9±60.4	n.s.	n.s.	n.s.
HDCA	41.2±5.6	4.9±3.5	29.4±9.0	↓:<0.001	↑:<0.01	↓:<0.05
HCA	4.5±0.7	13.7±6.3	4.2±0.8	n.s.	n.s.	n.s.
Total CDCA	1,148.5±147.7	45,196.7±9,624.4	2,509.6±647.7	↑:<0.001	↓:<0.001	↑:<0.01
Total CA	594.0±114.0	110,882.0±27,143.0	850.0±163.0	↑:<0.001	↓:<0.001	n.s.
Total DCA	677.8±106.1	651.8±151.9	523.6±132.2	n.s.	↓:<0.001	n.s.
Total LCA	59.7±6.9	27.8±4.1	101.2±22.4	↓:<0.01	↑:<0.001	n.s.
Total primary	1,743.0±226.0	156,079.0±33,461.0	3,359.0±741.0	↑:<0.001	↓:<0.001	↑:<0.01
Total Secondary	737.6±108.8	679.6±154.5	624.8±145.4	n.s.	n.s.	n.s.
Total 6α-Hydroxylated	45.6±5.7	18.7±7.5	33.6±8.9	↓:<0.001	↑:<0.01	↓:<0.01
Total Free	1,021.1±193.6	318.8±199.9	1,722.7±575.8	↓:<0.001	↑:<0.001	n.s.
Total Glyco	1,267.0±186.0	85,185.0±18,501.0	2,719.9±670.0	↑:<0.001	↓:<0.001	↑:<0.01
Total Tauro	373.2±73.0	71,364.6±17,253.0	744.0±145.0	↑:<0.001	↓:<0.001	↑:<0.001
TOTAL	2,669.0±316.0	156,873.0±33,526.0	5,201.0±1,111.0	↑:<0.001	↓:<0.001	↑:<0.001

Bile acids were quantified from 100 µL of serum from 40 healthy subjects (20 ♀ and 20 ♂) and 17 stenosed patients (8 ♂ and 9 ♀) before and after an endoscopic stenting of the bile duct. Bile acid levels are expressed in nM. *P*-values were determined by the Wilcoxon/Mann-Whitney rank-sum test; n.s.: not significant. CA, cholic acid; CDCA, chenodeoxycholic acid; DCA, deoxycholic acid; GCDCA, glycochenodeoxycholic acid; GCA, glycocholic acid; GDCA, glycodeoxycholic acid; GLCA, glycolithocholic acid; HCA, hyocholic acid; HDCA, hyodeoxycholic acid; LCA, lithocholic acid; LCA-S, LCA-sulfate; TCDCA, taurochenodeoxycholic acid; TCA, taurocholic acid; TDCA, taurodeoxycholic acid; TLCA, taurolithocholic acid; TUDCA, tauroursodeoxycholic acid; UDCA, ursodeoxycholic acid; TOTAL, sum of all bile acids.

On the other hand, the conjugated species of CDCA were also found to accumulate in samples from pre-stenting patients (138- and 37-fold increases in GCDCA and TCDCA levels, respectively; Table 1). These changes in CA and CDCA species led to a highly significant (*p*<0.001) accumulation of total primary acids (91-fold), total CA (186-fold) and total CDCA (39-fold) (Table 1). Other significantly increased species were TUDCA (15-fold) and TDCA (4-fold), while circulating levels of TLCA, GCA, GDCA and HCA were not significantly affected. Interestingly, unconjugated species of primary acids either remained unchanged (CA) or were even significantly reduced (CDCA). UDCA (85%; p<0.001), LCA (88%; p<0.001), LCA-S (42%; p<0.01), DCA (94%; p<0.001) and HDCA (88%; p<0.001) levels were also significantly reduced.

**Figure 3:**
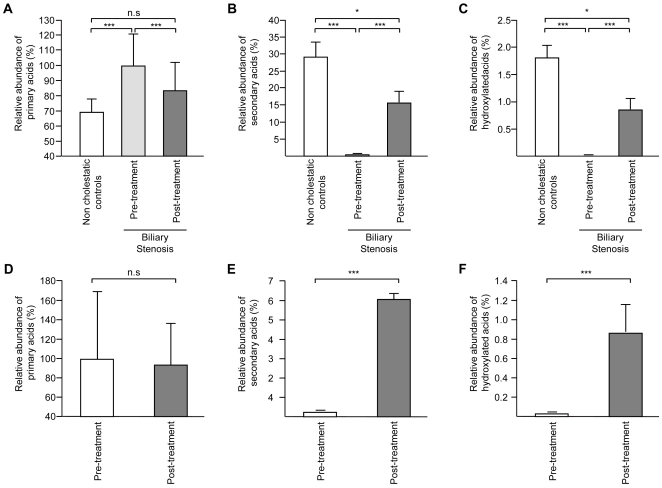
Relative abundance of primary (A & D), secondary (B & E) and 6α-hydroxylated (C & F) bile acids in serum (A–C) and urine (D–F) from non-cholestatic donors and/or patients before and after biliary stenting. Bile acids were quantified as indicated in the “Experimental Procedures” section. The relative abundance (expressed as a percentage) of primary, secondary and 6α-hydroxylated bile acid species was determined by adding up all unconjugated and/or conjugated species of CDCA+CA, LCA+DCA or HDCA+HCA, respectively, and by dividing the result by the total bile acids. Graphics represent the mean ± SEM (nM). *P*-values were determined by the rank sums Wilcoxon/Mann-Whitney test. *: *p*<0.05, ***: *p*<0.001. n.s.: not significant.

These observations demonstrate that biliary obstruction causes a drastic accumulation of primary bile acid species, and particularly of TCA and GCA. Consequently, the relative abundance of the primary species increased from 66 to 99.5% in sera from control donors and patients, respectively (Figure 3A). By contrast, the relative abundance of secondary and 6α-hydroxylated acids was significantly reduced (Figure 3B and C).

### Biliary stenting improves the circulating bile acid profile in patients with biliary obstruction

Biliary stenting almost completely corrected the bile acid profile abnormalities described above. In particular, TCA and GCA levels felt from 54.5±13.4 and 56.2±15.7 to 0.3±0.08 and 0.5±0.1 µM, respectively (Table 1). Consequently, after stenting, the relative abundance of these 2 species was significantly reduced to percentages similar to those observed in non-cholestatic donors (Figure 2). Post-stenting reductions were also observed for GCDCA and TCDCA (93% and 98%, respectively), thus leading to a strong reduction in total primary (97%), total taurine (99%) and total glycine (97%) levels (Table 1 and Figure 1). Almost all other species found to be altered in cholestatic samples underwent a correction as observed in samples obtained after stenting. Despite the striking changes observed, the serum concentrations of some acids remained significantly different from control levels after stenting (Table 1 and Figure 2). This was especially the case for TCDCA, GCDCA, GCA, TUDCA, GLCA and DCA, which remained higher in post-treatment samples as compared to non-cholestatic volunteers. As a result, total circulating bile acids remained 1.9-fold higher (Figure 1A) in post-treatment samples relative to non-cholestatic controls, while similar significant variations were also observed for total CDCA, total primary, as well as total glycine- and total taurine-conjugated species (Table 1). Except for the latter variations in absolute concentrations, the relative proportions of taurine- and glycine conjugated species in post-stenting sera were not significantly different from those detected in non-cholestatic samples (Figure 1B&C). The same also applies to the relative abundance of primary, secondary and 6α-hydroxylated acids (Figures 3A–C).

Overall, these observations indicate that biliary stenting is efficient at restoring an almost normal circulating bile acid profile.

### Biliary stenting also affects the urinary bile acid profile in stenosis patients

As for serum, the most abundant bile acid species in the urine from stenosis patients corresponded to the amidated forms of CA and CDCA (i.e. TCA, GCA, TCDCA and GCDCA, Table 2). However, almost all acids were found at lower concentrations in urine than in serum, with the notable exception of CA, HCA and HDCA concentrations, which were in the same range in both fluids (Tables 1&2).

**Table 2:** Urinary bile acid profile in cholestatic patients before and after stenting procedure.

	Pre-stenting	Post-stenting	
Bile acids	Mean ± SEM	Mean ± SEM	Change:*p*-value
CDCA	15.3±10.6	5.8±1.6	n.s.
TCDCA	3,396.6±3,316.7	30.2±14.6	↓:<0.001
GCDCA	8,628.3±8,307.0	80.5±31.8	↓:<0.01
CA	115.8±52.9	117.5±47.9	n.s.
TCA	7,361.9±5,295.1	234.6±155.4	↓:<0.001
GCA	10,177.2±4,654.6	487.5±254.4	↓:<0.001
UDCA	4.2±1.5	60.1±29.9	n.s.
TUDCA	181.8±55.9	49.5±37.4	↓:<0.01
LCA	BLD	BLD	n.s.
TLCA	0.7±0.7	BLD	n.s.
GLCA	0.9±0.6	0.8±0.5	n.s.
LCA-S	8.5±3.4	22.9±10.9	n.s.
DCA	2.9±1.1	11.2±2.5	↑:<0.01
TDCA	7.7±3.1	3.9±2.7	n.s.
GDCA	45.2±18.4	23.3±8.8	n.s.
HDCA	1.7±0.9	7.8±2.5	↑:<0.05
HCA	5.3±4.5	1.0±0.8	n.s.
Total CDCA	12,040.2±11,623.0	116.6±47.0	↓:<0.01
Total CA	17,654.9±9,435.9	839.6±411.5	↓:<0.001
Total DCA	55.8±20.6	38.3±11.9	n.s.
Total LCA	10.1±3.5	23.7±11.1	n.s.
Total primary	29,695.0±20,660.0	956.2±446.0	↓:<0.001
Total Secondary	65.9±22.2	62.0±18.1	n.s.
Total 6α-Hydroxylated	6.9±5.2	8.9±3.0	n.s.
Total Free	145.2±66.8	203.5±57.2	n.s.
Total Glyco	18,851.6±12,187.0	592.1±286.0	↓:<0.001
Total Tauro	10,948.6±8,594.1	318.2±206.5	↓:<0.001
TOTAL	29,953.9±20,661.0	1,136.7±492.0	↓:<0.01

Bile acids were quantified from 100 µL of urine from 17 stenosed patients (8 ♂ and 9 ♀) before and after an endoscopic stenting of the bile duct. Bile acid levels are expressed in nM. *P*-values were determined by the Wilcoxon/Mann-Whitney rank-sum test; n.s.: not significant. BLD, below the limit of detection. CA, cholic acid; CDCA, chenodeoxycholic acid; DCA, deoxycholic acid; GCDCA, glycochenodeoxycholic acid; GCA, glycocholic acid; GDCA, glycodeoxycholic acid; GLCA, glycolithocholic acid; HCA, hyocholic acid; HDCA, hyodeoxycholic acid; LCA, lithocholic acid; LCA-S, LCA-sulfate; TCDCA, taurochenodeoxycholic acid; TCA, taurocholic acid; TDCA, taurodeoxycholic acid; TLCA, taurolithocholic acid; TUDCA, tauroursodeoxycholic acid; UDCA, ursodeoxycholic acid; TOTAL, sum of all bile acids.

Restoring bile flow with stenting also resulted in a significant reduction of total urinary bile acids, which mainly reflected the decrease in TCDCA, GCDCA, TCA and GCA concentrations, respectively (Table 2). Urinary levels of other species such as TUDCA, DCA and HDCA were also significantly altered by stenting, but only to a lower extent (Table 2). The relative abundance of primary, secondary and 6α-hydroxylated acids followed a similar pattern as in serum, with a modest reduction of primary acids (from 99.7 to 93%) and a more pronounced induction of secondary and 6α-hydroxylated forms (Figures 3D–F).

These results indicate that biliary stenting also affects the urinary elimination of bile acids.

## Discussion

The present study provides the first comprehensive analysis of the profiles of circulating and urinary bile acids during bile duct obstruction and after restoration of bile flow via biliary stenting. According to previous reports [2], [8], [14], a strong accumulation in total circulating bile acids is observed in patients with biliary stenosis. We also observed an almost complete restoration of the bile acid profile in serum samples after stenting, while the same treatment strongly reduced urinary bile acids. Of note, the reduction in serum acids is close to values previously reported in comparable extrahepatic cholestasis populations [2], [14]. In addition to these original comparative analyses, an important novel aspect of the current work is the species-specific manner in which circulating and urinary acids are affected by both bile flow interruption and restoration. Indeed, of the 17 acids quantified, 6 underwent a significant increase in concentration, 5 were not significantly altered, while 6 were significantly reduced in sera from biliary obstruction patients as compared to control samples (Table 1). Most of the acids undergoing reductions in levels corresponded to secondary and 6α-hydroxylated species, whereas the sole primary acid within this group was CDCA. Interestingly, the reduction in CDCA levels is in accordance with the previously reported downregulation of the cytochrome P450 (CYP)7A1, the rate-limiting bile acid synthesizing enzyme, in the liver of patients with biliary obstruction [2]. This effect was suggested to be a response of the liver to bile duct obstruction aimed at reducing the production of toxic bile acids [2]. However, such an outcome would be only partly reached since circulating levels of conjugated primary acids were dramatically increased. The latter observation indicates that a reduction in CYP7A1 expression is insufficient to abolish the production of bile acids during biliary obstruction.

In addition to reducing CYP7A1, biliary obstruction also upregulates CYP8B1 expression in the liver [2]. This gene encodes sterol 12α-hydroxylase, a key branch in the bile biosynthetic pathway which favors CA instead of CDCA formation, and thus determines the CA/CDCA ratio [16]. Accordingly, this ratio increased from 0.70 in control samples to 2.05 in sera from biliary obstruction patients in the current study. This increase mainly reflects the differential behavior of serum CA and CDCA levels: i.e. CDCA was significantly reduced, while CA remained almost unchanged. A similar increase also occurred when considering the total CA/total CDCA ratio (0.51 and 2.45 in control and cholestatic samples, respectively). As expected, biliary stenting was efficient in reducing these ratios. These results indicate that the production of CA instead of CDCA is favored during biliary obstruction and that restoring the bile flow reinstates the preferential conversion of cholesterol into chenodeoxycholic acid species. In addition to their synthesis, the relative abundance of CA and CDCA species may also be affected by their urinary elimination. In urine, both pre- and post-stenting levels of CA (free or conjugated) are higher than those of CDCA (Table 2). These results indicate that CA derivatives are more efficiently excreted in urine than those of CDCA. However, the fact that the stenting procedure had a more profound impact on CDCA suggests that urinary elimination of CDCA and its conjugates improved during biliary obstruction.

These differential variations in abundance of primary bile acid species are thought to affect the cytotoxic properties of the total bile acids. Indeed, CA presents a lower hydrophobicity and higher critical micelle concentration than CDCA [13]. As a result, CA and its conjugates display lower cytotoxicity at high concentration (reviewed in Perez MJ and Briz O [13]). Thus, the preferential accumulation of CA instead of CDCA species supports the hypothesis that bile duct obstruction triggers an adaptive response aimed at limiting bile acid cytotoxicity [13].

A strong increase in serum TCA levels occurs in serum from stenosed patients (Table 1). Similar TCA accumulations were previously observed in other cholestatic situations, such as intrahepatic cholestasis of pregnancy [17], thus supporting the possible role of TCA as a biomarker for altered biliary circulation [17]. This TCA accumulation is representative of the stronger increase of circulating levels of taurine-conjugated bile acids as compared to that of glyco-bile acids. Such a difference may also interfere with the toxicity of the serum bile acids since taurine conjugates, which can activate cell survival and anti-apoptotic pathways, are less toxic than their glycine counterparts [18], [19], [20], [21]. This is particularly the case for TUDCA [22], [23], [24], [25], which is 15-fold higher in stenosed than control sera.

In contrast to primary bile acids, total secondary species remained at comparable levels in all serum samples, and their relative abundance was therefore drastically reduced in both serum and urine from pre-operative cholestatic patients (Figure 3). Interestingly, circulating levels of various species, such as LCA, LCA-S and DCA, were significantly reduced during biliary obstruction as compared to controls (Table 1). These observations are in accordance with the reported absence of LCA accumulation in the liver of patients with obstructive jaundice [26]. More importantly, secondary acids are indicators of the entero-hepatic recirculation of bile acids since they are formed in the intestine via the enzymatic dehydroxylation of primary acids catalyzed by bacterial enzymes [4]. The reduction in serum LCA and DCA levels is therefore a consequence of the strong impairment of the entero-hepatic bile acid recirculation in biliary stenosis patients. Surprisingly, circulating levels of TDCA, the dehydoxylated metabolite of TCA, were significantly higher in cholestatic than in control sera. A likely explanation for this unexpected observation is that a small amount of TCA, which undergoes an enormous accumulation in the liver sustains enzymatic or non-enzymatic dehydroxylation in hepatic cells. On the other hand, secondary acids such as LCA are hydrophobic molecules and among the most toxic bile acids [10], [27]. It is therefore remarkable that biliary obstruction results in a strong decrease in the relative abundance of these toxic species.

In conclusion, profiling circulating and urinary bile acids in biliary stenosis patients before and after stenting procedures reveals that in addition to the dramatic increase of their total concentration in both fluids, each species is differentially affected. Furthermore, the stronger accumulation of less toxic species further supports the notion that biliary obstruction activates a series of self-defense mechanisms aimed at protecting the organism against the inherent toxicity of bile acids [2]. Finally, we also showed that biliary stenting is efficient in restoring an almost normal circulating bile acid profile.

## Materials and Methods

### Ethics Statement

This study was approved by the appropriate clinical study review boards at the CHUQ Research Centre, Laval University (“*Comité d’éthique de la recherche Clinique du CHUL*”, Québec, QC, Canada: projects #95.05.14 and #97.05.14) and the Pomeranian Medical University (Bioethics commission, Pomeranian Medical University in Szczecin, Poland: resolution N° BN-001/43/06). All patients had signed a written consent form before each procedure.

### Materials

Unconjugated and taurine and glycine-conjugated bile acids were purchased from Steraloids (Newport, RI). Deuterated bile acids (d4-CA, d4-CDCA, d4-LCA and d4-DCA) were purchased from C/D/N Isotopes (Montréal, Canada). Protein assay reagents were obtained from Bio-Rad Laboratories Inc. (Marnes-la-Coquette, France). HPLC-grade solvents were from VWR Canlab (Montréal, QC, Canada). Solid-phase extraction (SPE) columns used were 60 mg/3 mL Strata-X 33 µm polymeric reversed-phase from Phenomenex (Torrance, CA).

### Non-cholestatic volunteers and patients with biliary obstruction

Liver biochemistries from non-cholestatic volunteers (control) and subjects with biliary obstruction before and after biliary stenting are shown in Table 3.

**Table 3:** Liver biochemistries of patients involved in the study.

	Normal	Non-cholestatic	Stenosed patients	Stenosed patients
	Range	Subjects	Before stenting	After stenting
AST (U/L)	5–43	29.40±1.32	219.67±36.12&&&	33.30±6.28***
ALT (U/L)	5–60	26.13±1.99	290.17±55.04&&&	44.90±12.03*** &
AP (U/L)	20–140	n.d.	715.23±189.91	157.10±23.73***
γGT (U/L)	5–80	n.d.	1154.58±225.31	238.90±91.74**
Total bilirubin (mg/dl)	0.1–1.2	0.54±0.04	15.06±1.65&&&	1.60±0.32*** &&&

Liver biochemistries were determined in 40 non-cholestatic subjects (20 ♀ and 20 ♂) and 17 stenosed patients (8 ♂ and 9 ♀) before and after an endoscopic stenting of the bile duct. Values represent the mean ± SEM. *P*-values were determined by the Wilcoxon/Mann-Whitney rank-sum test.

**: *p*<0.01 vs. before treatment,

***: *p*<0.001 vs. before treatment,

& : *p*<0.05 vs. non-cholestatic,

&&& : *p*<0.001 vs non-cholestatic. ALT: alanine aminotransferase; AST: aspartate aminotransferase; AP: alkaline phosphatase, γGT: γ-glutamyl transpeptidase; n.d.: not determined.

Serum samples from non-cholestatic donors (20 men and 20 women) were selected within the participants to the Genetics of Lipid Lowering Drugs and Diet Network (GOLDN) study who were recruited exclusively at the Minnesota fields center (Minneapolis, MN), a single-arm, uncontrolled, nonrandomized intervention aimed at identifying genetic factors associated with interindividual variability of the triglyceride response to high-fat meals and fenofibrate [28], [29], [30], [31]. Only participants who had not taken lipid-lowering agents for at least 4 weeks before the initial visit were included. Exclusion criteria were as reported [28], and included elevated lipids and serum concentration of liver enzymes (aspartate aminotransferase and alanine aminotransferase) [28]. As extensively described in Lai et al. [28], participants took part in five visits. However, sera used in the present study were drawn at the first visit, when patients were free of any drug treatment or lipid challenge.

Seventeen patients (8 men and 9 women; mean age 64±10 years) with clinical and biochemical features of cholestasis were recruited. In all cases, dilatation of the biliary tree was first confirmed with abdominal ultrasound. Final clinical diagnoses in patients included were as follows: common bile duct stones, 8 patients; pancreatic tumor, 5 patients; bile duct tumor, 1 patient; benign common bile duct stenosis, 1 patient and chronic pancreatitis, 1 patient. The endoscopic retrograde cholangiopancreatography (ERCP) procedure involved placement of plastic 10F stents which were removed or replaced at a median of 5 weeks after initial procedure, depending on the diagnosis.

### Bile acid measurements

Bile acid concentrations were determined using a HPLC-MS/MS system with an electrospray interface, using a novel method adapted from Ye et al. [32]. The modified method allows the simultaneous evaluation of 17 species in the same sample (Figure 1A). Solid-phase extraction (SPE) was initiated by adding 2 mL of a 0.1% (w/v) formic acid solution and 30 µL of internal standards (i.e: the deuterated bile acids d4-CDCA, d4-CA, d4-LCA and d4-DCA) to 100 µL of serum. The same treatment was applied to analytical standards which were diluted (1∶1) with 100 µL of adsorbed serum, and subsequently used to generate calibration equations. SPE columns were conditioned with 1 mL MeOH and 2 mL of 0.1% formic acid. Columns were successively washed with 2 mL of H_2_O and 2 mL of H_2_O∶MeOH (80∶20) containing 0.1% formic acid under negative pressure. Bile acids were eluted with 2 mL of MeOH. Eluates were completely evaporated at 45°C under N_2_ and reconstituted in 100 µL of H_2_O∶MeOH (50∶50) containing 5 mM ammonium acetate and 0.01% formic acid. Fifteen µL of sample or calibration standards were then injected into the LC-MS/MS system.

A single LC method was used for the separation of the free, taurine, glycine and sulfate conjugates of bile acids. The chromatographic system consisted of an Alliance 2690 Separations Module (Waters, Milford, MA). Analytes were separated using a 50×3 mm Synergi Hydro-RP column (2.5-µm particles) (Phenomenex, Torrance, CA). The chromatographic conditions used were: 5 mM ammonium acetate-0.01% formic acid in water (solvent A), 5 mM ammonium acetate-0.01% formic acid in MeOH (solvent B), and acetonitrile (solvent C) at a flow rate of 800 µL/min. The chromatographic program was as follows: (i) initial conditions: 40% A: 55% B: 5% C for 4 min; (ii) a linear gradient to 80% B was applied over the next 8 min; (iii) the column was flushed with 90% B for the next 2 min; and (iv) re-equilibration to the initial conditions over the next 4 min. All analytes were quantified by tandem mass spectrometry (MS/MS) using an API3200 LC/MS/MS instrument (Applied Biosystems, Concord, ON, Canada). The analytical run required only 15 min, and the lower limit of detection varied from 2.2 (LCA-S) to 8.0 nM (TCDCA).

### Data analysis

Baseline characteristics as well as bile acid concentrations were evaluated as means ± SEM for continuous variables. The total bile acid concentration corresponds to the sum of the 17 bile acid levels. The sums of glyco- and tauro-conjugates were calculated by adding up the concentrations of conjugated CDCA, CA, DCA and LCA. The sum of free (i.e. unconjugated) bile acids also includes HDCA and HCA levels. Total levels of primary, secondary and 6α-hydroxylated bile acid species were determined by adding up all unconjugated and/or conjugated species of CDCA+CA, LCA+DCA or HDCA+HCA, respectively. The relative abundance of a given species or group of species was calculated as its concentration divided by the total bile acid concentration, and is expressed as a percentage of total bile acids. Bile acid concentrations were not normally distributed according to the Shapiro-Wilk test, and therefore, the Wilcoxon/Mann-Whitney rank-sum test was used instead for statistical purposes. Correlations were assessed by the Spearman's rank correlation coefficient using the JMP Statistical Discovery V7.0.1 program (SAS Institute, Cary, NC).
